# Characterizing COVID-19 waves in urban and rural districts of India

**DOI:** 10.1038/s42949-022-00071-z

**Published:** 2022-10-28

**Authors:** Bhartendu Pandey, Jianyu Gu, Anu Ramaswami

**Affiliations:** 1 Department of Civil and Environmental Engineering, Princeton University, Princeton, NJ 08540 USA; 2 National Renewable Energy Laboratory, 15013 Denver West Parkway, Golden, CO 80401 USA

**Keywords:** Environmental studies, Geography

## Abstract

Understanding spatial determinants, i.e., social, infrastructural, and environmental features of a place, which shape infectious disease is critically important for public health. We present an exploration of the spatial determinants of reported COVID-19 incidence across India’s 641 urban and rural districts, comparing two waves (2020–2021). Three key results emerge using three COVID-19 incidence metrics: cumulative incidence proportion (aggregate risk), cumulative temporal incidence rate, and severity ratio. First, in the same district, characteristics of COVID-19 incidences are similar across waves, with the second wave over four times more severe than the first. Second, after controlling for state-level effects, urbanization (urban population share), living standards, and population age emerge as positive determinants of both risk and rates across waves. Third, keeping all else constant, lower shares of workers working from home correlate with greater infection risk during the second wave. While much attention has focused on intra-urban disease spread, our findings suggest that understanding spatial determinants *across* human settlements is also important for managing current and future pandemics.

## Introduction

With over 55% of the global population currently living in urban areas^1^, understanding the urban disease burden is important. The physical proximity of people, as well as infrastructure provisioning in urban areas (such as water and sanitation), have historically shaped endemics, epidemics, and pandemics^2^. Increased physical interactions between people due to higher mobility within and across urban areas nationally and globally have furthered the influence of these urban characteristics on infectious diseases^3–5^. Against this backdrop, understanding the spatial determinants of infectious diseases at urban and intra-urban scales becomes particularly important. Recent scientific studies suggest a significant role of urban infrastructure, socioeconomic stratifications, population density, and other key urban characteristics on COVID-19 transmission^6–8^. Analyzing several types of human settlements—populated places and regions—in a country can yield insights into how human settlement variations shape COVID-19 transmission.

This study focuses on COVID-19 incidence in India across its urban and rural districts, motivated by three main reasons. First, India is the second-most populous country in the world, with over 1.3 billion people. Second, India is at the cusp of urbanization, i.e., expected to significantly urbanize between now and the middle of this century, with its demographic base rapidly shifting from rural to urban via natural urban population growth, rural-to-urban population migration, rural land reclassification, and urban land expansion^9,10^. Significant spatial variations in population density, infrastructure, built environments, and socioeconomic features characterize the country’s transitory phase. Some of these variations contribute to comorbidities and shape health disparities within and across regions and urban areas. Third, COVID-19 transmission in India is particularly understudied from a sub-national perspective, with most studies focusing on national- or state-level data^11–14^. Some studies have reported significant spatial and temporal heterogeneities underlying COVID-19 transmission in India, which necessitate understanding disease progression and associated spatial determinants across human settlements along the rural-urban continuum. COVID-19 transmission waves observed in India^15^ present a significant opportunity to compare the two dominant waves in the context of their spatial determinants. Such comparisons can advance our understanding of how urbanization and associated variations shaped COVID-19 incidence across waves.

Recent studies conducted in India have highlighted place-based characteristics, which may have made some parts of India more vulnerable to COVID-19 transmission than others. The greater relative severity of the second wave in India compared to the first wave—in terms of reported incidences and mortalities—demonstrated several additional vulnerabilities, such as a low level of health care access and overall health care infrastructure deficit^16,17^. Studies focusing on finer spatial scales (district-level) identified several key determinants of, and vulnerabilities to, COVID-19 transmission. For instance, population density is closely associated with the spatial distribution of COVID-19 cases in India^18–20^. This finding contrasted with the distribution of COVID-19 cases in the United States, where population density had no association with COVID-19 incidence rates in the initial phases of disease spread when the size of the metropolitan areas had been controlled for^21^. Besides density, urbanization rates (share of urban population to the total population) also determine COVID-19 transmission risk in India^22^, owing to greater social interactions in more urbanized areas compared to others. Similarly, out-migration from cities also explains the spread of COVID-19^23^. These associations are consistent with basic predictions from meta-population epidemiological models^4,5^.

The overarching goal of this paper is to examine if there are a common set of spatial determinants, i.e., characteristics of a place, whose variations across districts shaped disease incidence across the two waves of COVID-19 transmission in India. Specifically, we focus on urbanization and attendant spatial variations in social-infrastructural-environmental dimensions, as a determinant of COVID-19 incidence characteristics across districts in India. Furthermore, we examine COVID-19 incidence severity during the second wave relative to the first wave across districts (Table 1). To accomplish these goals, we assembled a dataset that brings together social, ecological, infrastructural, and urban form characteristics along with data on COVID-19 incidences across 641 districts (the administrative equivalent of counties in the US) spanning the entire rural-to-urban continuum in India. Such integrative data frameworks incorporate multiple social determinants of health and have been applied in public health^24^ and sustainability science^25^.

**Table 1:** Cumulative COVID-19 metrics analyzed in this study using COVID-19 incidence data across the two dominant waves.

S. No.	Cumulative COVID-19 metrics	Description
1.	Cumulative Incidence Proportion	A measure of aggregate incidence risk in each wave
2.	Cumulative Temporal Incidence Rate	A measure of the rapidity of COVID-19 incidence in each wave
3.	Severity Ratio (cumulative proportion)	A measure of incidence risk severity in wave 1 compared to wave 2
4.	Severity Ratio (cumulative rate)	A measure of incidence rate severity in wave 1 compared to wave 2

Our analysis complements compartmental models—such as the Susceptible, Exposed, Infected, and Recovered (SEIR) model—that focus on epidemic curves under different model parameters. These compartmental models focus primarily on temporal dynamics but can be integrated with spatially explicit demographic, socioeconomic, infrastructural, and environmental data to understand disease progression across space and time. This requires an understanding of the key determinants of disease incidence, which can complement, inform, and help validate such integrative models. This study seeks to evaluate whether there are spatial determinants whose variations across districts shape cumulative incidence patterns based on four metrics (Table 1). We explore the spatial determinants of COVID-19 cumulative incidence proportion (*CIP*), and their overall impacts on disease risk across waves. In addition, to explore the spatial determinants underlying rapid COVID-19 transmission, we use cumulative temporal incidence rate (*CIR*) as a second metric. Lastly, we evaluate severity ratios (*SR*) comparing these metrics between the second (more severe) wave relative to the first wave: *SR*_*CIP*_ *and SR*_*CIR*_. We control for state-level effects (such as mask mandates, data collection capacities, and other relevant policies) that might impact *CIP*, *CIR*, and *SR* at the district level.

## Results

### COVID-19 metrics across waves

**Fig. 1:**
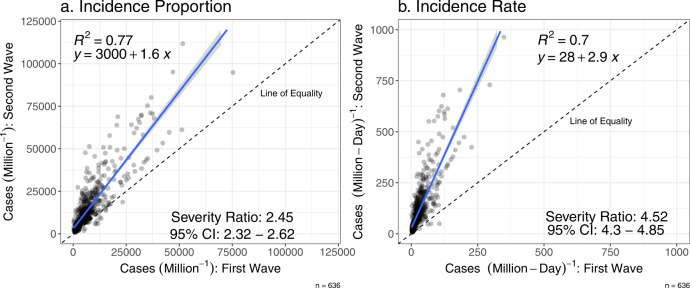
Higher COVID-19 incidences in the second wave compared to the first wave, on average. Comparing COVID-19 (**a**) cumulative incidence proportion (per one million people) and (**b**) cumulative incidence rate (per one million person-day), between the first (*x* axis) and the second (*y* axis) wave. The dashed line indicates the line of equality, and the solid blue line represents linear regression fit. COVID-19 incidence proportions and rates were on average higher in the second wave than in the first wave. The panels also show the estimated average severity ratio and 95% confidence interval based on bootstrap estimations with 100,000 replications. Spearman’s correlation coefficients between the first and second wave are 0.87 and 0.86, for cumulative incidence proportions and rates, respectively, and significant at the 0.01 level.

Our results suggest that COVID-19 transmission across waves in India may be associated with a common set of spatial determinants, whose variations across districts shape disease incidence. Results show that the spatial variations of cumulative incidence proportions and rates are consistent across waves, on average (Fig. 1). For instance, compared to other districts, a district with a higher incidence (in terms of incidence proportion or rate) in the first wave also had a higher incidence in the second wave. Spearman’s correlation coefficient between cumulative incidence proportions (rates) for the first and second wave is 0.87 (0.86) and significant at the 0.01 level. This spatial consistency suggests similar infection drivers across waves in India.

**Fig. 2:**
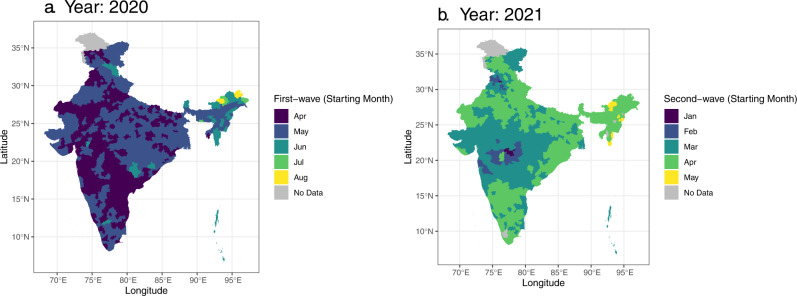
COVID-19 onset timing across districts. Estimated COVID-19 wave onset month for the first (**a**) and the second (**b**) wave across districts. Districts with no data are filled with gray color. Spatial patterns of onset months for the first wave (left) highlight multiple leading districts, whereas the second-wave (right) patterns emphasize a few concentrated leading districts.

Analysis of starting dates for each wave suggests two leading centers during the second wave. Such specific centers do not appear in the first wave (Fig. 2a). We estimate the initial onset timing of April 2020 in 243 districts (of 639 districts) during the first wave, suggesting multiple COVID-19 leading centers in the first wave. In contrast, we identify 27 districts (of 638 districts) with an initial onset timing of February 2021 during the second wave located predominantly in the state of Maharashtra and Punjab (Fig. 2b). These second wave leading centers encompass two tier-1 cities (Mumbai and Pune) and several tier-2 cities (Amritsar, Aurangabad, Chandigarh, Indore, and Jalandhar Jalgaon, Nagpur, and Patiala). Reports from India corroborate these findings.

Comparing COVID-19 cumulative temporal incidence rates between the two dominant waves and across districts, we find that the second COVID-19 wave was over four times more severe than the first wave, on average. We estimate an average *SR* metric of 4.52 across districts with a 95% confidence interval of 4.3–4.85 (Fig. 1b). Furthermore, we find significant (*P* value < 0.01) spatial autocorrelation in relative second-wave severity across districts with a Moran’s I index of 0.31, suggesting an underlying spatial structuring. The severity ratios also emphasize the role of geography in infection spread at the regional scale (Supplementary Figs. 1 and 2). We estimate ~37% (of 636) districts with *SR* greater than the average but with the greatest second-wave severity in four northeastern states (Meghalaya, Mizoram, Manipur, and Sikkim). The lower physical access in northeastern states and associated limited inter-district population movement in relation to other states can explain the lower COVID-19 incidence during the first wave and delayed COVID-19 spread, i.e., higher infections in the second wave.

Consistent with reports of greater infection risk in the second wave than the first wave due to the Delta variant, we find that *R*_*0*_ $\left( {R_t^\mu } \right)$ was 1.2 (1.22) times higher in the second wave than the first wave, suggesting greater contagiousness (Supplementary Fig. 3). However, our results show insignificant spatial consistency (across districts) between the two waves in *R*_*0*_ and $R_t^\mu$ estimates (*P* value > 0.1).

### Correlation and regression analysis

After accounting for state-level differences, results underscore aggregate development, based on urbanization and wealth, as a determinant of COVID-19 infection risk and spread. Urbanization rate and wealth (a measure of living standards) correlate positively with COVID-19 incidence proportion and rate. Global Moran’s I indices indicate significant (*P* value < 0.01) spatial autocorrelation in COVID-19 incidence proportions ($I_1^p = 0.53$ and $I_2^p = 0.50$) and rates ($I_1^r = 0.47$ and $I_2^r = 0.52$), across the two waves. This makes OLS estimates biased and inconsistent. Based on spatial lag and spatial error models and accounting for state-level differences, we find that a unit increase in urbanization is associated with a 1.2% (1.4%) increase in incidence proportion in the first (second) wave across districts (Table 2 and Supplementary Tables 1 and 3). Similarly, we find a 1.2% (1.3%) increase in incidence rate in the first (second) wave across districts (Table 2 and Supplementary Tables 2 and 3). Regression estimates also show significant positive associations for wealth with incidence proportions and rates across the two waves. Urbanization rate and wealth, in many ways, can be considered a measure of aggregate development. By extension, COVID-19 incidence proportion and temporal incidence rate tend to increase with increasing aggregate development levels across districts in India.

**Table 2:** Regression estimates of the effect size of varied spatial characteristics on cumulative incidence proportions (*CIP*) and incidence rates (*CIR*).

Variable	Log(*CIP*) first wave	Log(*CIP*) second wave	Log(*CIR*) first wave	Log(*CIR*) second wave
Urbanization	0.012^***^ (0.002)	0.014^***^ (0.002)	0.012^***^ (0.002)	0.013^***^ (0.002)
Wealth	0.703^***^ (0.100)	0.460^***^ (0.095)	0.631^***^ (0.095)	0.436^***^ (0.096)
Log (population density)	−0.116^**^ (0.055)	−0.151^***^ (0.039)	−0.134^**^ (0.052)	−0.160^***^ (0.038)
Population age	0.056^***^ (0.019)	0.083^***^ (0.016)	0.045^**^ (0.019)	0.081^***^ (0.016)
Log (no travel)	−0.198^*^ (0.116)	−0.318^***^ (0.105)	−0.152 (0.117)	−0.274^***^ (0.106)
Log (distance)	−0.082 (0.106)	0.029 (0.087)	−0.101 (0.101)	0.019 (0.088)
Log (1+in-degree (1st wave))	0.023^*^ (0.012)		0.019 (0.012)	
Log (1+in-degree (2nd wave))		0.006 (0.010)		0.004 (0.009)
Constant	8.014^***^ (0.715)	7.012^***^ (0.627)		2.206^***^ (0.630)
λ	0.28***	0.25***	0.31***	0.29***
Observations	636	639	636	639
State fixed-effects	Yes	Yes	Yes	Yes
Log-likelihood	−420.830	−364.894	−405.872	−368.335
Akaike information Criterion	927.660	815.788	897.743	822.670
Pseudo-R^2^	0.784	0.797	0.784	0.790

^*^P, ^**^P, ^***^P < 0.01; standard errors are heteroskedasticity-corrected.

Results suggest a greater risk of disease spread in districts with a lower share of the population working from home, but only during the second wave. *Ceteris paribus*, a 1% increase in the percentage share of workers working from home is associated with a 0.32% (0.27%) decrease in incidence proportion (rate) during the second wave. We find this association insignificant for the first wave, at 0.05 significance level, suggesting the role of strict nationwide lockdown during the first wave in shaping these associations as well as differences between the waves. Furthermore, we consistently find insignificant associations with average commute distance and inter-district population movement (based on the in-degree metric; see “Methods”). These results align with weak (*ρ* ≤ 0.2) or insignificant correlations (*P* value > 0.01) between incidence metrics and in-degree metrics (Supplementary Table 4), even after the in-degree metrics were normalized by the length of the associated wave.

Results also show a consistent positive association between incidence proportions and rates and average population age, across waves. We estimate that a 1-year increase in average population age is associated with 5.6% (8.3%) increase in incidence proportion and 4.5% (8.1%) increase in incidence rates, during the first (second) wave (Table 2 and Supplementary Tables 1–3). Greater elasticity for the second wave compared to the first wave emphasizes that districts with a greater older population were more vulnerable to COVID-19 transmission than other districts, especially in the second wave.

**Fig. 3:**
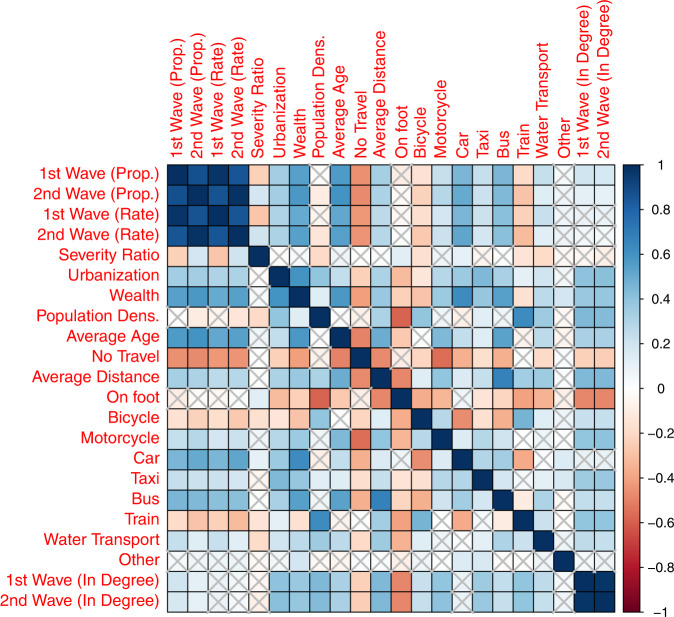
Correlations between COVID-19 incidence metrics and relevant social, ecological, infrastructural, and urban form characteristics. Correlation matrix denoting Spearman’s correlation coefficients estimated for COVID incidence proportions and rates, severity ratio, urbanization, wealth (denoting living standards), population density, average age, commute to work variables, and in-degree estimates across districts (*n* = 636).

Between population density and incidence proportions, and incidence rates across waves, we find a small and consistently negative association, *ceteris paribus*. Our correlation analysis suggests a weak (during the second wave) and insignificant (during the first wave) association between population density and incidence proportion and rates (Fig. 3). However, our regression estimates suggest a negative association once other variables are held constant (Table 2). We find that a 1% increase in population density incidence proportions (rates) decreased by 0.16% (0.13%) during the first wave and by 0.15% (0.16%) during the second wave (Table 2). These findings are counter-intuitive but contribute to the debate surrounding the relevance of population density in contributing to greater incidence risk.

Regression models also suggest that districts with lower wealth levels had greater incidence severity in the second wave compared to the first (Supplementary Table 5). Similarly, districts with older population, on average, had greater second-wave severity. Our estimates show that a 1-year increase in average population age was associated with a 3.6% increase in second-wave incidence severity relative to the first, based on incidence rates. These results corroborate with our findings from comparing the associations between individual waves. Overall, they highlight that the second wave was more severe in underserved districts as well as districts with older population.

## Discussion

As highlighted in a recent United Nations report^26^, COVID-19 has (re)emphasized rethinking urban form and function. Statistically significant associations between the spatial determinants and COVID-19 incidence identified in the present study underscore the importance of urbanization and associated spatial characteristics vis-à-vis public health in India. Here, our results suggest that planning a pandemic-proof urban transition in India will require a spatial understanding of its inter-urban characteristics (across human settlements) in addition to an intra-urban focus.

Consistent with the first law of geography, results show a significant spatial structuring across districts in India, i.e., COVID-19 cumulative incidence in each district is associated with the incidence in adjoining districts. This finding is consistent with previous reports concerning cumulative COVID-19 cases and mortalities across districts^27,28^. However, our results show spatial structuring in incidence proportions and temporal incidence rates across the two waves and in relative second-wave severity. This significant spatial structuring underscores COVID-19 transmission as also a regional process in India.

In the context of spatial determinants, we find similar COVID-19 incidence characteristics between waves and across districts. This suggests that some geographies are inherently more vulnerable to COVID-19 incidences than others, which has implications for future COVID-19 waves and other infectious diseases of similar characteristics. Prioritizing these geographies for disease control and prevention can reduce the disease burden more effectively. To this end, our findings put forth urbanization, wealth, population age, and population density as the significant spatial determinants of COVID-19 incidence. Disease control and prevention nationally, as a result, can be more effective by prioritizing districts based on these characteristics. With the impending urban transition, our results suggest that such prioritization may become even more critical in the context of future epidemics and pandemics of similar characteristics. In the contemporary context, results suggest that advancing epidemiological modeling on COVID-19 in India can benefit from incorporating geographic variations in social, economic, and infrastructural dimensions. In this direction, a shift from aspatial to spatial modeling will be necessary. However, our findings with respect to the negative association of population density need further investigation, considering proxies of super spreader events or population activity densities.

Our results also highlight some differences in incidence characteristics across waves. First, the second wave in India was over four times more severe on average than the first wave, based on temporal incidence rate-based severity ratio. This greater second-wave severity can be attributed to less-stringent lockdown in the second wave than the first wave^12,29^ and more transmissible viral variants^30^. Across districts, we find significant differences in the severity ratio between states, but only average wealth and population age explained district-level variations. Second, we find characteristic differences in the leading centers of disease spread across the two waves. These differences suggest that the spatial patterns of disease onset tend to be less predictable and emphasize the importance of continued disease surveillance across districts in India. Third, we find that % workers working from home only explain spatial variations in COVID-19 incidence proportions and rates during the second wave, when lockdowns were less stringent compared to those imposed during the first wave. In contrast, commute distance had an insignificant association once other factors were controlled for. Existing studies have reported associations between the temporal patterns of human mobility and COVID-19 outcomes^31–33^. However, our analysis did not find inter-district population movement as a key spatial determinant of incidence proportion and rate.

Our study highlights several areas that need further investigation. First, our study design is sensitive to baseline population data. In the absence of a recent census dataset, future studies can analyze the implications of using different population data sources, each generated with a unique set of assumptions, on incidence risk assessment. Second, our interpretation of the associations assumes that state-level differences control for potential bias in COVID-19 incidence reporting. Although this assumption is consistent with significant disparities in data quality across states in India^34^, it is yet unclear how data quality varies across districts. Third, while we limited the scope of the present study to reported COVID-19 incidences owing to reporting concerns with respect to mortality^35,36^, it is unclear whether similar associations exist with COVID-related mortalities in India. Fourth, our study shows an insignificant association between social media platform-derived inter-district population movement data and COVID-19 incidences, suggesting inter-district population movement as an insignificant spatial determinant across districts in India. Still, these datasets could help us understand COVID-19 incidences vis-à-vis human mobility within districts and across time^37^, requiring an in-depth spatiotemporal analysis of movement data vis-à-vis COVID-19 incidences. Fifth, our study focuses on basic epidemiological metrics that capture patterns of disease incidence but contributes little towards a detailed understanding of transmission dynamics over time and under different COVID-19 variants.

Overall, our study emphasizes that our understanding of the spatial determinants of COVID-19 incidence will benefit substantially from investigations into the geographic characteristics and scale of human settlements. While much attention has focused on intra-urban disease spread, our findings suggest that understanding spatial determinants across human settlements is also important in managing current and future pandemics. Databases such as the one developed and analyzed here can advance a foundational understanding of the spatial determinants of human health relevant for future pandemics.

## Methods

### Data

#### COVID-19 data

We use daily district-level COVID-19 reported incidence data (until July 31, 2021, based on the waning of the second wave) from the COVID19India.org platform, a citizen-science-based data collection and validation effort, which has compiled incidences, recoveries, deaths, and vaccination data from various government and media reports. We preprocess the data to ensure nationwide coverage and spatial consistency with Census data. District-level data is unavailable for a few States and Union Territories (Assam, Sikkim, Manipur, Telangana, Delhi, Goa, and Andaman & Nicobar Islands). Consequently, we disaggregate the data for these regions using a population-weighted allocation of daily incidence, informed by our observation of significant correlations between (log) cumulative incidence and (log) population size (Pearson’s correlation coefficient = 0.73) and recent scaling analyses applied to COVID-19 incidences^38–40^.

#### Demographic and urban boundaries data

We use the population dataset for 2020 from WorldPop with age groupings, owing to the absence of a recent Census dataset and given higher spatial resolution (~100 m)^41^ compared to other candidate datasets such as, Landscan^42^ and GPW^43^. We aggregate this dataset (using zonal statistics) to estimate total population, population density, and average population age, at the district level. In addition, we use 2018 Global Human Settlement Layer Dataset (https://ghsl.jrc.ec.europa.eu/, at 10 m) to identify urban areas (probability threshold = 0.1) and derive urban population shares (% urban) by combining with the WorldPop dataset.

#### Census data

We use the 2011 Census (https://censusindia.gov.in) as the data source for modes of travel to work (as a share of the working population commuting by a specific mode of transportation) and commuting patterns across districts in India. We focus on two variables for commuting patterns: the share of the working population with no commute. We calculate % workers working from home for each district as a percentage share of the number of workers with “No Travel” to the total working population. For the average commuting distance to work, we take the mid-point of the distance bins with maximum distance of 60 km). Owing to the absence of a recent dataset, we assume that the cross-sectional distributions of these characteristics remain invariant between 2011 and the temporal coverage of the COVID-19 time series.

#### Social media data

We use Facebook’s population movement data^44^ available at the district level and an 8-h frequency to estimate the size of the incoming population during the duration of the wave, based on in-degree metric (*in_degree*_*j*_) for each edge, i.e., district (*j*), of the network with *V* vertices (Eq. (1)).

1 $$in\_degree_j = \mathop {\sum}\nolimits_{v \in V} {w_{j \leftarrow i}(v_{j \leftarrow i})}$$

#### Wealth data

District-level income or wealth data in India has limited availability. The existing national consumer expenditure surveys (CES) measure consumption-expenditure as an income proxy, which is less reliable at the district level due to the limited sample size. This study uses a household assets-based measure of wealth or living standards from the 2015–16 demographic and health survey (DHS) with an approximated six times greater sample size than CES, with 601,509 sampled households. Available as a composite index at the household level (https://dhsprogram.com/), we estimate weighted district-level average wealth index, using sampling weights.

### COVID-19 metrics across waves

#### COVID wave characterization

**Fig. 4:**
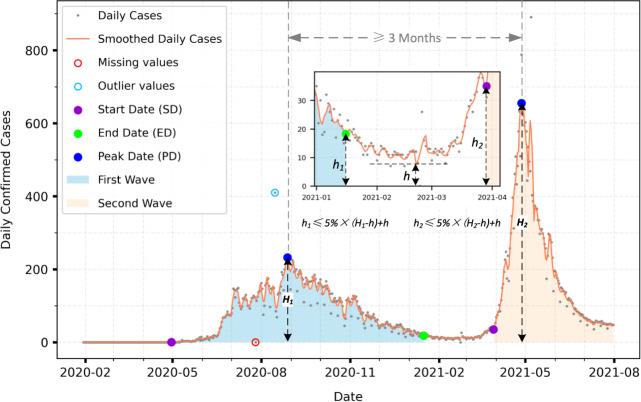
COVID-19 wave characterization. Illustrating COVID-19 data processing and waves characterization.

Before characterizing the waves, we use two data processing steps: preprocessing and wave metrics estimation. Preprocessing detects outliers, interpolates missing values, and filters the time series. Outlier detection identifies anomalous values within a 14-day window based on a 10% (P_1_) and 90% (P_2_) percentile range (PR): *PR* = *P*_2_ – *P*_1_. We define outliers as values falling below *P*_1_ – 3*PR* or above *P*_2_ + 3*PR* and linearly interpolate the outliers and other missing values within the 14-day window (Fig. 4). To further reduce noise, we use a Savitzky–Golay (SG) filter, which filters the time series while preserving the shape and height of the curve based on least-squares polynomial approximation. SG filter has two critical parameters: window size and order of the polynomial. After trial and testing, we note that using small temporal window sizes and large polynomial order retains noise in the time series. Consequently, we select 14 days as the optimal window size and a second-order polynomial in the SG filter. During the wave metrics estimation, we focus on three metrics: peak date (*PD*_*i*_), start date (*SD*_*i*_), and end date (*ED*_*i*_) across *i* waves. We detect *PD*_*i*_ based on the following criterion: a peak’s amplitude is greater than the third quartile of the incidence time series and any two local peaks are three months apart. Following the peak date detection, we estimate the starting and ending dates for the waves. For the first wave, we identify the start date as to when the changes in the number of confirmed cases turn to a positive integer. For the subsequent waves, we identify the start date such that its amplitude *h*_2_ meets the following condition (Eq. (2)).

2 $$h_2 \le 5{{{\mathrm{\% }}}} \times (H_2 - h) + h$$

3 $$h_1 \le 5{{{\mathrm{\% }}}} \times (H_1 - h) + h$$

*H*_2_ is the peak’s amplitude of the non-first wave (Fig. 4). *h* is the lowest amplitude between this peak and the previous peak (Fig. 4). The start date of this non-first wave is defined as the first date on which the value for (*h*_*2*_*–h*) is equal to or less than 5% of (*H*_*2*_*–h*) searching backward from this wave’s peak. The end date of any wave was determined using the same heuristic as the starting date of a non-first wave (Eq. 3). The difference was that an end date was the first date whose value (*h*_*1*_*–h*) is equal to or less than 5% of (*H*_*1*_*–h*) searching forward in time from this wave’s peak.

Our wave detection and characterization algorithm yielded two (three) COVID-19 waves for 610 (26) districts, out of 639 districts (Supplementary Fig. 4). Of the 26 districts, we find districts encompassing two cities—Delhi and Ahmedabad—and others relatively rural, i.e., urbanization rates less than 50%. As the national- and state-level aggregated data stress two COVID-19 waves, our algorithm underscore the importance of understanding patterns at finer spatial scales, where novel dynamics can be at play. For example, in Delhi, we estimate three waves but with a bi-modal distribution during the first wave period of the country. In other districts, we observe that a relatively lower number of cases, with a speckle signature, during the beginning of the pandemic led to anomalous wave detection. Consequently, we assume the first wave comprises the estimated first two waves in districts with estimated three waves.

Building on the outputs of our algorithm, we focus on three metrics^45^: (1) cumulative incidence proportion, (2) temporal incidence rate, and (3) severity ratio. Cumulative incidence proportion (*CIP*_*ij*_) is a measure of disease risk defined here as a ratio of cumulative COVID-19 incidence (*I*_*ij*_) during a wave (*i*) over total population (*Pop*_*j*_) for a district (*j*) (Eq. (4)).

4 $$CIP_{ij} = \frac{{\mathop {\sum }\nolimits_{t = 1}^t I_{ij}}}{{Pop_j}}$$

Cumulative temporal incidence rate (*CIR*_*ij*_) is a measure of the rapidity of disease incidence amongst a population and over a fixed time period (*t* days) per wave (*i)* (Eq. (5)).

5 $$CIR_{ij} = \frac{{\mathop {\sum }\nolimits_{t = 1}^t I_{ij}}}{{t \ast Pop_j}}$$

Finally, we introduce a severity ratio metric (*SR*_*j*_) to measure the severity of the second COVID-19 wave in India compared to the first wave (Eq. (6)).

6 $$SR_j = \frac{{CIR_{2j}}}{{CIR_{1j}}}$$

As a preliminary analysis, we analyze spatial variations in *CIP*_*ij*_, *CIR*_*ij*_, and *SR*_*j*_. First, we compare *CIP*_*ij*_, and *CIR*_*ij*_, between the two dominant waves and across districts to examine spatial consistency in disease risk and rates of disease spread. Next, we analyze the spatial patterns of COVID-19 wave start date for the two waves to identify leading centers. Finally, we analyze *SR*_*j*_ metric across districts and estimate the average second-wave severity (relative to the first wave) and associated 95% confidence intervals based on bootstrap estimation with 100,000 replications^46^. We noted a significant correlation between severity ratio metrics calculated with incidence proportions and incidence rates with a Pearson’s correlation coefficient of 0.91. Consequently, we use incidence rates to examine second-wave severity here and in our subsequent correlation and regression analysis.

Beyond these metrics, we estimate R_0_ based on exponential growth rate^47,48^, with a gamma distribution for the serial interval, assuming a mean and standard deviation of 4.4 and 3 days, respectively, for the generation time^49^. Using the same serial interval assumptions, we estimate R_t_ using a maximum-likelihood estimation^50^, but based on incidence curves based on start and peak dates of the wave, yielding an average estimate per wave $\left( {R_t^\mu } \right)$. We also report district-level average severity ratios based on estimated R_0_ and R_t_, computed with a functional form consistent with Eq. (6). However, owing to data quality concerns, assumptions underlying R_0_ and R_t_ estimations, and our focus on COVID-19 incidences in India, we restrict our detailed analysis to *CIP*_*ij*_, *CIR*_*ij*_, and *SR*_*j*_ (Supplementary Fig. 5 and Supplementary Table 6).

### Correlation and regression analysis

We use correlation and regression analysis to characterize general features of COVID-19 incidence across districts, spanning the entire rural-to-urban gradient. Specifically, we focus on the associations between COVID-19 incidence metrics and related key spatial characteristics, i.e., urbanization, population density, wealth, and mobility (Supplementary Table 6 and Supplementary Fig. 6). First, we explore non-linear monotonic associations between COVID-19 incidence metrics and spatial characteristics by examining Spearman’s correlations. In the process, we identify key urban features relevant for COVID-19 incidences. We further examine these associations using ordinary least squares (OLS) and, after examining the presence of spatial autocorrelation, using spatial regression models (estimated with *spatialreg package*^51^ in *R*), i.e., spatial lag models (SLM) and spatial error models (SEM). These spatial models account for potential bias and inconsistencies in OLS estimates due to spatial autocorrelations^52^ (Eqs. (7) and (8)). In our analysis, we control for state-level differences, to account for reporting bias^34^, and interpret the model with the largest log-likelihood estimate and lowest Akaike Information Criterion (AIC) metric between OLS, SLM, and SEM. Finally, we report Bayesian Markov Chain Monte Carlo estimates^51^ for the selected spatial models, as a robustness check.

7 $$Y = \rho \left( W \right)y + \beta \left( X \right) + \varepsilon$$

Where, *Wy* spatially lagged outcome variable for spatial weights matrix *W, ε* is the error term, and *ρ* and *β* are the model parameters.

8 $$Y = \beta \left( X \right) + \lambda \left( W \right)\varepsilon + \upsilon$$

Where, *ε* is the spatial autocorrelated error terms, *υ* are independently and identically distributed errors, and *λ* and *β* are the model parameters.

### Ethical approval

In consultation with the office of the Institutional Review Board (IRB), Human Research Protection Program at Princeton University, we determined that an ethical approval was not required for our research based on the human subjects research definition and scope.

## Supplementary information

Supplementary Information

## Data Availability

This study used publicly available datasets, all of which are referenced under the Data section. COVID-19 reported incidence data is available from https://www.covid19india.org. Global Human Settlement Layer dataset used for urban areas delineation is available from https://ghsl.jrc.ec.europa.eu. Population data used in the study is available from https://hub.worldpop.org/geodata/summary?id=50436. District-level commuting patterns data is available from https://censusindia.gov.in. Social media platform-derived daily population movement data can be accessed from https://dataforgood.facebook.com. 2015–2016 Demographic and Health Survey (DHS) dataset from which we derive the district-level wealth index is available from https://dhsprogram.com. District-level harmonized datasets analyzed in this study are available from https://github.com/bhartendupandey/India_COVID19_Waves.git.

## Code availability

The underlying python and R code used to derive the findings are available from a publicly-accessible repository at https://github.com/bhartendupandey/India_COVID19_Waves.git.
