# Supplementary material for: Characterizing COVID-19 waves in urban and rural districts of India
Source: NPJ Urban Sustain. 2022 Oct 28;2(1):26. doi: 10.1038/s42949-022-00071-z (PMC9613454; doi:10.1038/s42949-022-00071-z)
Supplement: Supplementary file 1 — Supplementary Information [file 42949_2022_71_MOESM1_ESM.pdf]

Supplementary Information

Characterizing COVID-19 waves in urban and rural districts of India

Bhartendu Pandey[a*], Jianyu Gu[b,a], Anu Ramaswami[a¶]

[a] Department of Civil and Environmental Engineering, Princeton University, Princeton, NJ 08540, U.S.A.¶

[b] National Renewable Energy Laboratory,15013 Denver West Parkway, Golden, CO 80401, U.S.A.

* Corresponding Author

Email: bhartendu.pandey@princeton.edu

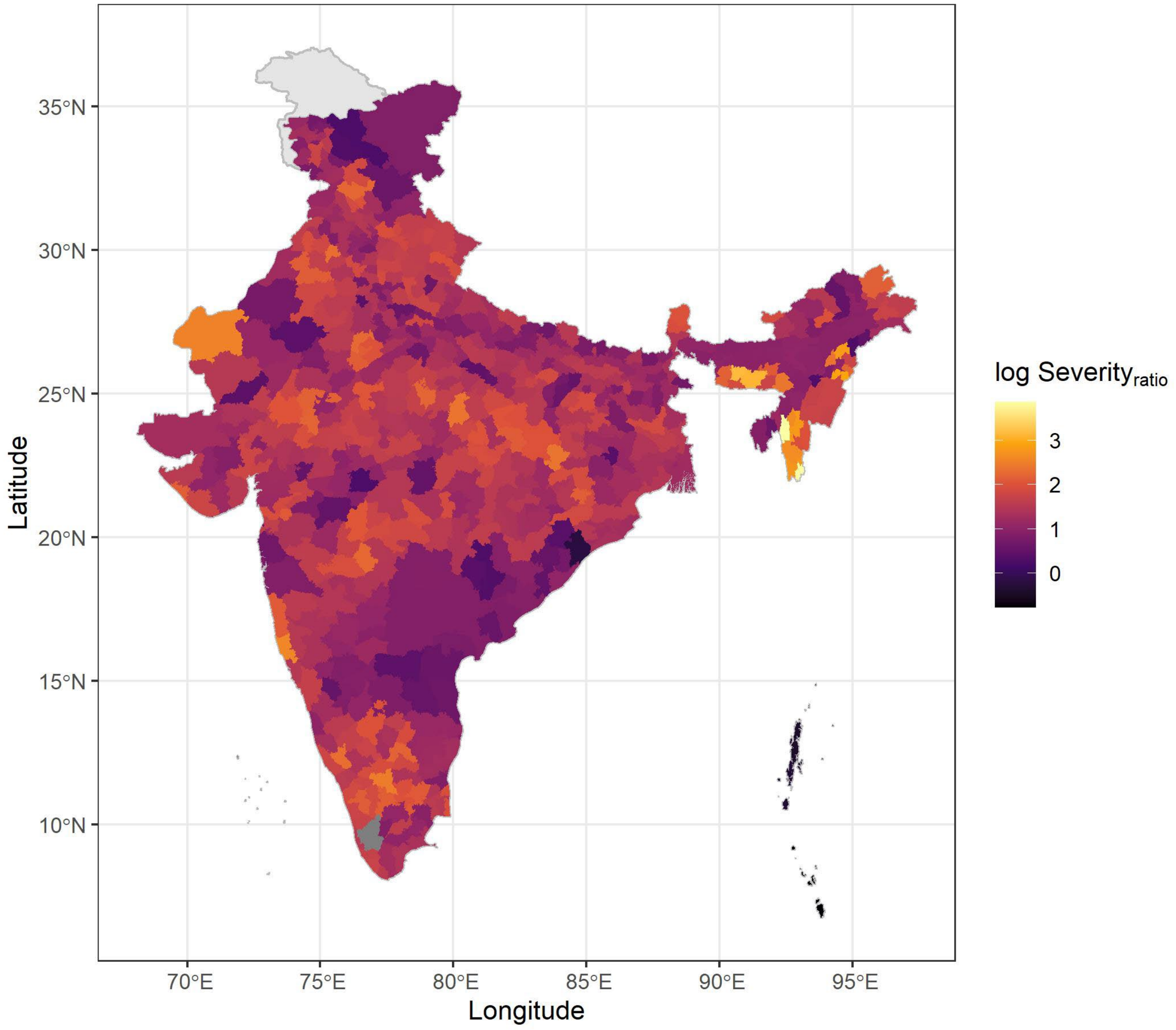

Supplementary Figure 1. Severity ratios across districts. Spatial distribution of (log) Severity Ratio (SR), based on cumulative temporal incidence rates, denoting second-wave severity relative to the first wave. Districts with no data are represented in gray color.

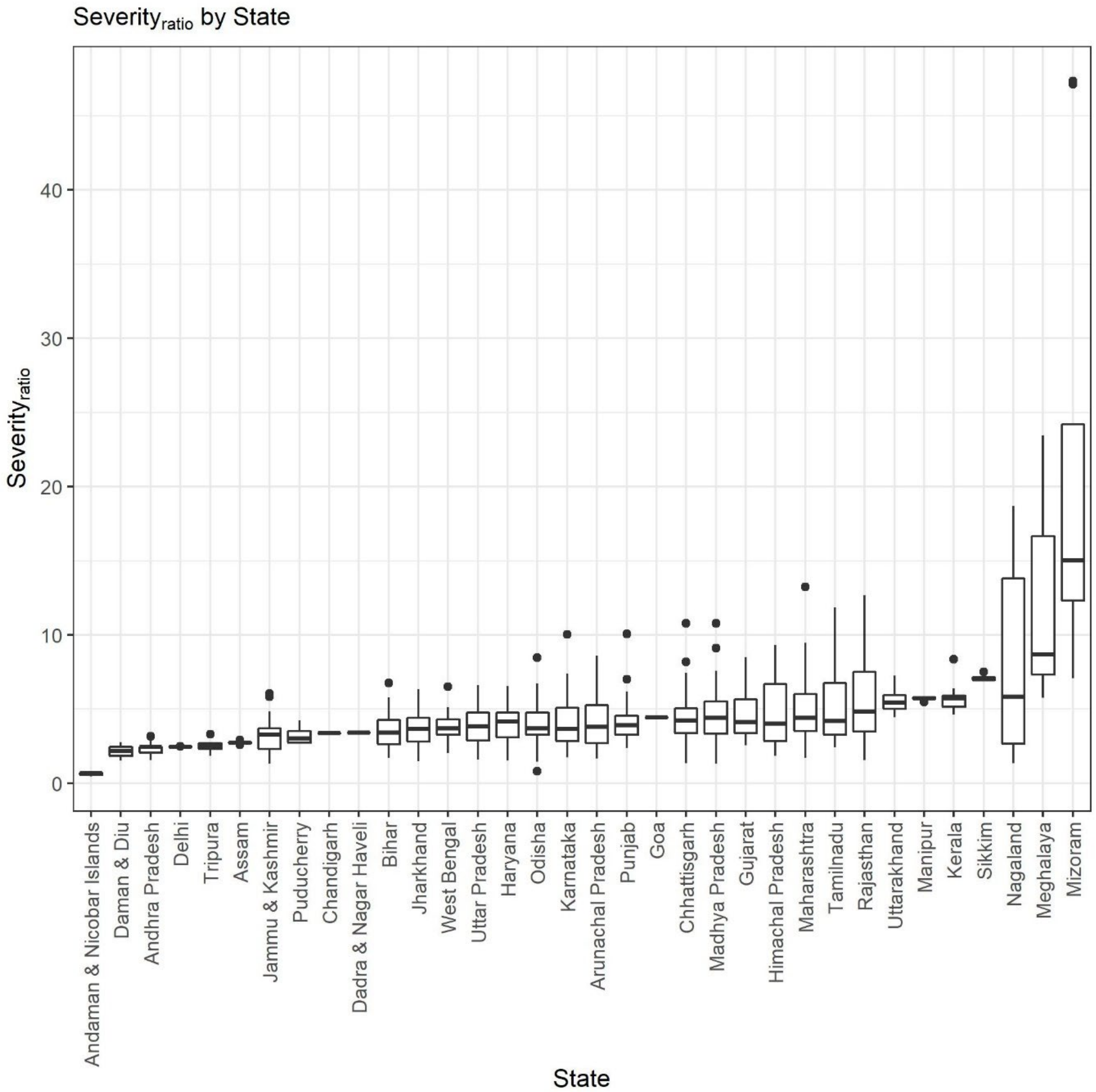

Supplementary Figure 2. Severity ratios across states. Distribution of Severity Ratio (SR) denoting second-wave severity relative to the first wave across states in India. Note that the state classification is as per 2011.

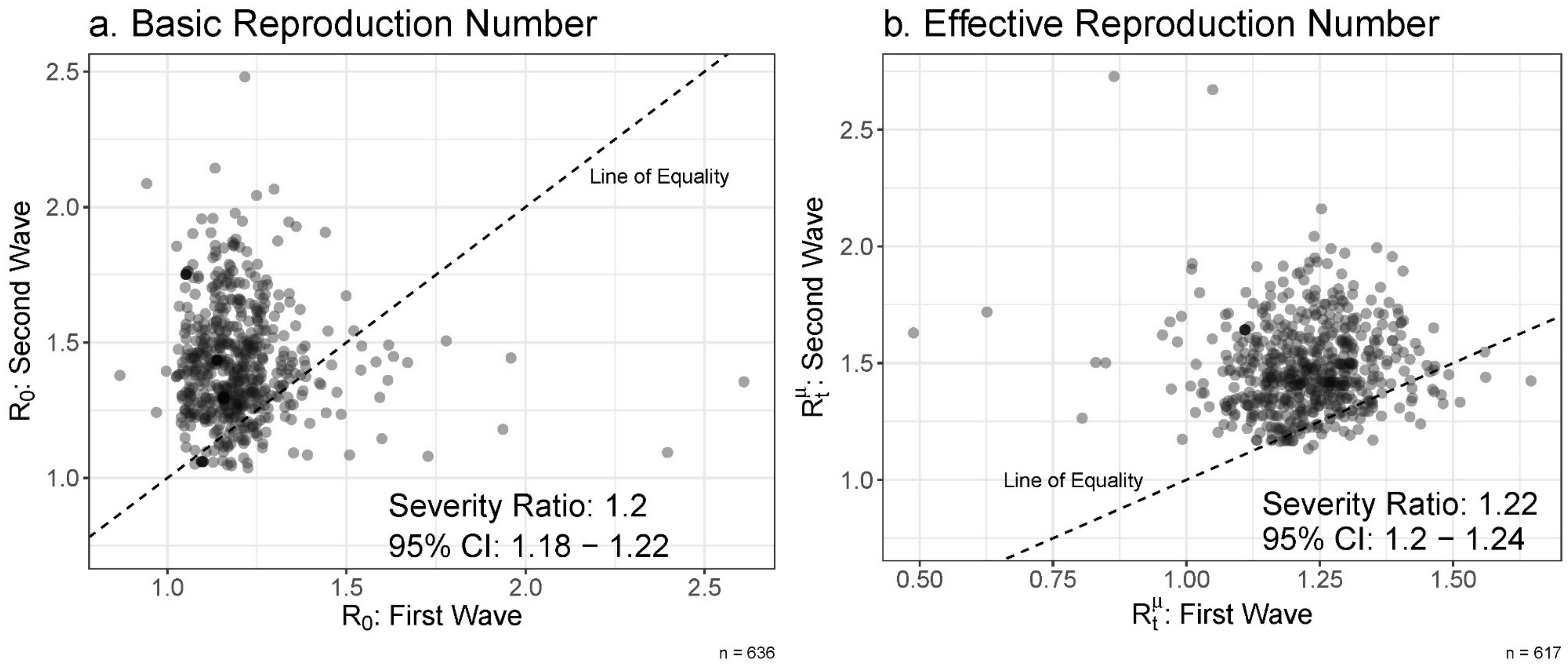

Supplementary Figure 3. Basic and Effective Reproduction numbers across waves. Comparing COVID-19 (a) basic reproduction number (R_0) and (b) average effective reproduction number (R_t^{μ}) between the first (x-axis) and the second (y-axis) wave. The dashed line indicates the line of equality, and the solid blue line represents linear regression fit. The panels also show the estimated average severity ratio and 95% confidence interval based on bootstrap estimations with 100,000 replications.

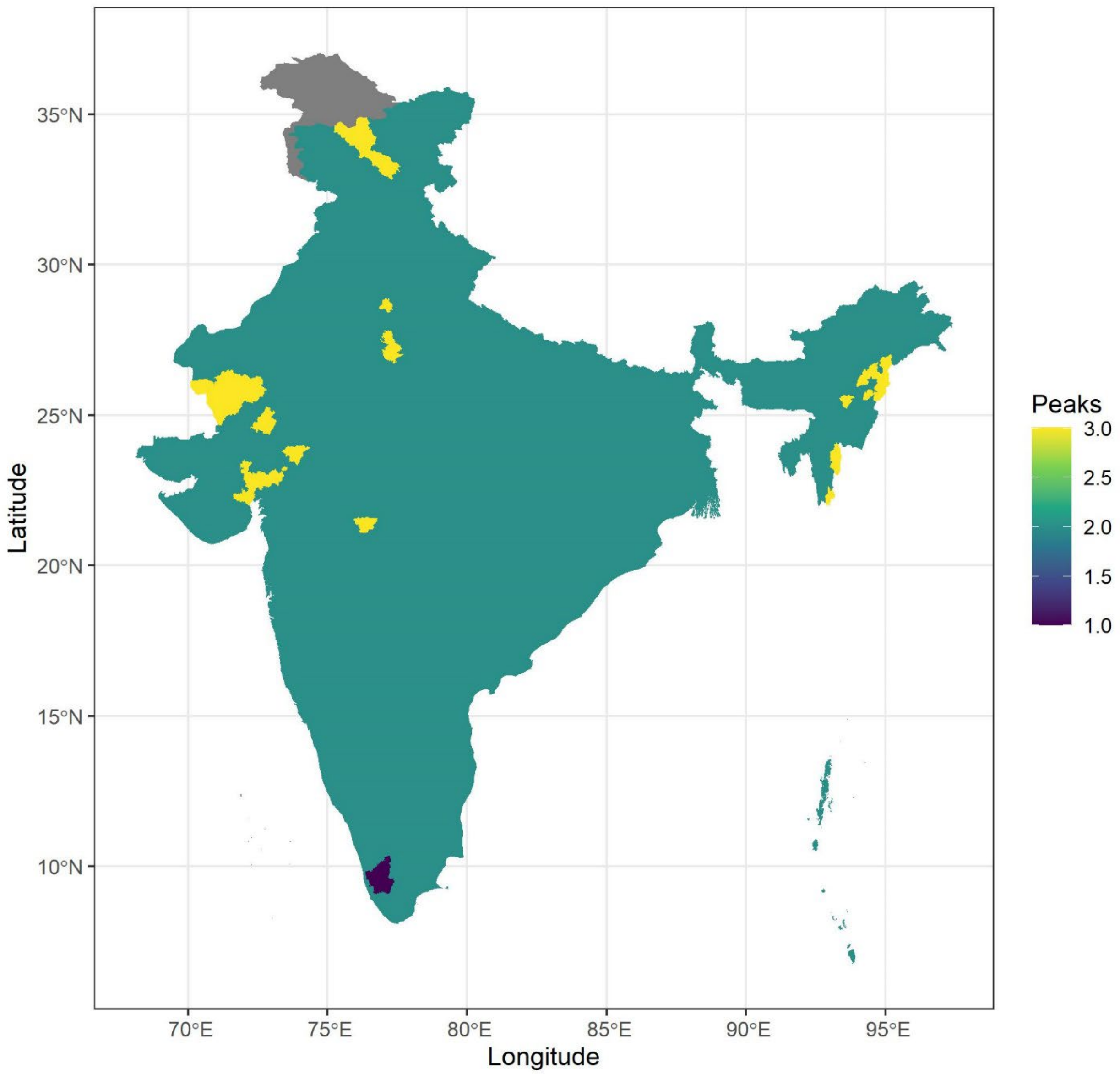

Supplementary Figure 4. Dominant two COVID-19 incidence waves across India. Map showing the estimated number of COVID-19 waves across districts in India. Districts shown in gray color have missing data.

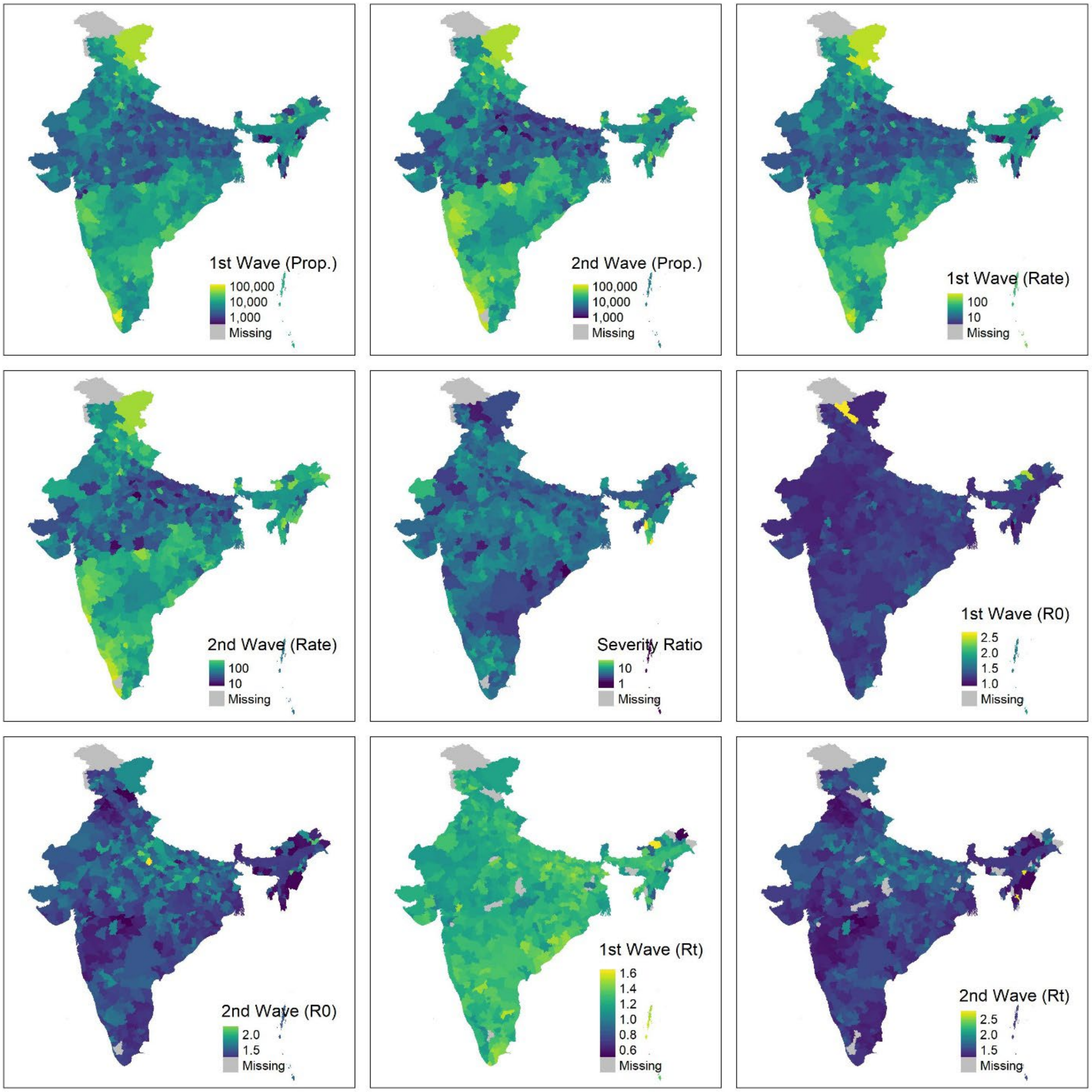

Supplementary Figure 5. COVID-19 metrics across districts. Spatial Distributions of COVID-19 metrics considered in the study.

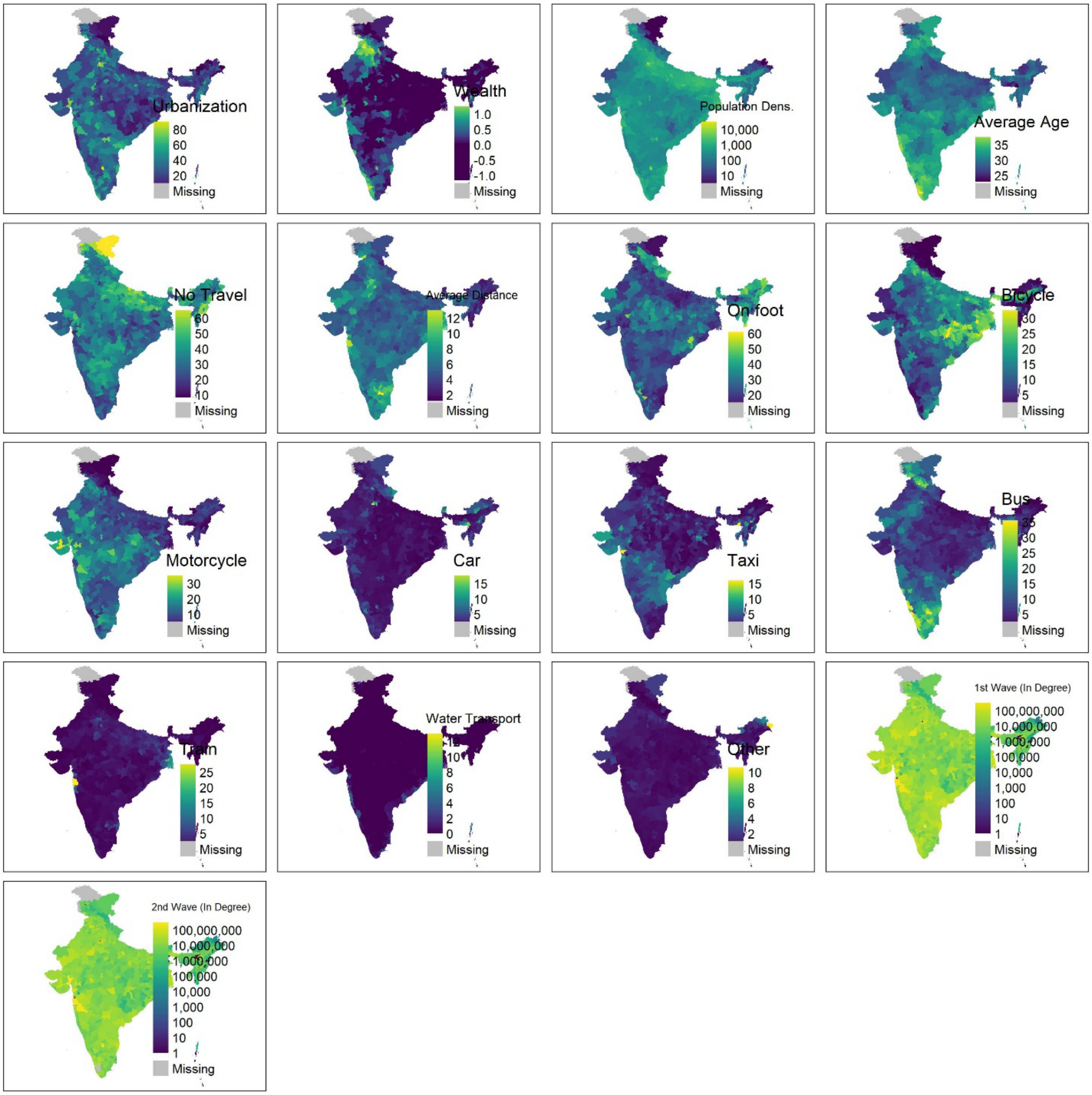

Supplementary Figure 6. Select COVID-19 covariates. Spatial Distributions of key covariates considered in the study.

Supplementary Table 1. Regression results for COVID-19 cumulative incidence proportions with state-level fixed effects.

	**Log(Cumulative Incidence Proportion)**					
		**First Wave**			**Second Wave**	
	(1) OLS	*(2) Spatial Lag*	*(3) Spatial Error*	*(4) OLS*	*(5) Spatial Lag*	*(6) Spatial Error*
Urbanization	0.011*** (0.002)	0.011*** (0.002)	0.012*** (0.002)	0.013*** (0.002)	0.013*** (0.002)	0.014*** (0.002)
Wealth	0.672*** (0.090)	0.647*** (0.090)	0.703*** (0.100)	0.426*** (0.089)	0.419*** (0.088)	0.460*** (0.095)
Log(Population Density)	-0.128** (0.054)	-0.123** (0.053)	-0.116** (0.055)	-0.146*** (0.037)	-0.144*** (0.037)	-0.151*** (0.039)
Population Age	0.054*** (0.017)	0.051*** (0.017)	0.056*** (0.019)	0.085*** (0.015)	0.084*** (0.015)	0.083*** (0.016)
Log(No Travel)	-0.194* (0.111)	-0.202* (0.111)	-0.198* (0.116)	-0.326*** (0.103)	-0.324*** (0.103)	-0.318*** (0.105)
Log(Distance)	-0.081 (0.100)	-0.098 (0.100)	-0.082 (0.106)	0.023 (0.084)	0.018 (0.084)	0.029 (0.087)
Log(1+In-Degree(1st Wave))	0.021 (0.013)	0.023* (0.013)	0.023* (0.012)			
Log(1+In-Degree(2nd Wave))				0.004 (0.009)	0.005 (0.009)	0.006 (0.010)
Constant	8.161*** (0.662)	8.269*** (0.658)	8.014*** (0.715)	6.988*** (0.607)	7.032*** (0.605)	7.012*** (0.627)
State Fixed effects?	Yes	Yes	Yes	Yes	Yes	Yes
Rho	-	0.08*	-	-	0.03	-
Lambda	-	-	0.28***	-	-	0.25***
Observations	639	639	639	636	636	636
R^2	0.778	0.779	0.784	0.792	0.792	0.797
Adjusted R^2	0.763			0.778		
Log Likelihood		-428.609	-420.830		-371.783	-364.894
sigma^2		0.224	0.215		0.188	0.182
Akaike Inf. Crit.		943.219	927.660		829.566	815.788
Residual Std. Error	0.490 (df = 598)			0.449 (df = 595)		
F Statistic	52.245*** (df = 40; 598)			56.618*** (df = 40; 595)		
Wald Test (df = 1)		3.151*	25.893***		0.441	19.449***
LR Test (df = 1)		3.073*	18.632***		0.429	14.207***
Note:	*p**p***p<0.01; Nagelkerke R^2 reported for spatial models; Standard errors are heteroskedasticity-corrected					

Supplementary Table 2. Regression results for COVID-19 cumulative incidence rates with state-level fixed effects.

	Log (Cumulative Incidence Rate)					
		**First Wave**			**Second Wave**	
	(1) OLS	*(2) Spatial Lag*	*(3) Spatial Error*	*(4) OLS*	*(5) Spatial Lag*	*(6) Spatial Error*
Urbanization	0.011*** (0.002)	0.011*** (0.002)	0.012*** (0.002)	0.012*** (0.002)	0.012*** (0.002)	0.013*** (0.002)
Wealth	0.607*** (0.086)	0.571*** (0.086)	0.631*** (0.095)	0.409*** (0.089)	0.392*** (0.088)	0.436*** (0.096)
Log(Population Density)	-0.146*** (0.051)	-0.137*** (0.050)	-0.134** (0.052)	-0.155*** (0.036)	-0.150*** (0.036)	-0.160*** (0.038)
Population Age	0.041** (0.017)	0.039** (0.017)	0.045** (0.019)	0.083*** (0.015)	0.079*** (0.015)	0.081*** (0.016)
Log(No Travel)	-0.153 (0.113)	-0.162 (0.112)	-0.152 (0.117)	-0.282*** (0.104)	-0.278*** (0.103)	-0.274*** (0.106)
Log(Distance)	-0.096 (0.096)	-0.119 (0.095)	-0.101 (0.101)	0.027 (0.085)	0.013 (0.084)	0.019 (0.088)
Log(1+In-Degree(1st Wave))	0.016 (0.013)	0.019 (0.013)	0.019 (0.012)			
Log(1+In-Degree(2nd Wave))				0.002 (0.009)	0.004 (0.009)	0.004 (0.009)
Constant	3.636*** (0.666)	3.744*** (0.657)	3.425*** (0.714)	2.174*** (0.612)	2.278*** (0.605)	2.206*** (0.630)
State Fixed effects?	Yes	Yes	Yes	Yes	Yes	Yes
Rho	-	0.12***	-	-	0.07	-
Lambda	-	-	0.31***	-	-	0.29***
Observations	639	639	639	636	636	636
R^2	0.776	0.778	0.784	0.784	0.784	0.790
Adjusted R^2	0.761			0.769		
Log Likelihood		-414.169	-405.872		-376.680	-368.335
sigma^2		0.213	0.205		0.191	0.183
Akaike Inf. Crit.		914.337	897.743		839.360	822.670
Residual Std. Error	0.481 (df = 598)			0.453 (df = 595)		
F Statistic	51.812*** (df = 40; 598)			53.846*** (df = 40; 595)		
Wald Test (df = 1)		7.006***	31.864***		2.642	26.856***
LR Test (df = 1)		6.735***	23.329***		2.563	19.253***

Note: *p**p***p<0.01; Nagelkerke R^2 reported for spatial models; Standard errors are heteroskedasticity-corrected

Supplementary Table 3. Bayesian estimates of spatial error models for COVID-19 cumulative incidence proportion (CIP) and rate (CIR) rates with state-level fixed effects.

Model	Wave	Variable	Estimate	Lower Bound	Upper Bound
**CIP**	Wave 1	Urbanization	0.01229	0.008027	0.01661
**CIP**	Wave 1	Wealth	0.71573	0.526003	0.90634
**CIP**	Wave 1	Pop. Dens.	-0.10989	-0.182368	-0.03698
**CIP**	Wave 1	Pop. Age	0.05762	0.020148	0.09481
**CIP**	Wave 1	No Travel	-0.19737	-0.446545	0.04116
**CIP**	Wave 1	Distance	-0.08005	-0.278486	0.11655
**CIP**	Wave 1	In-Degree	0.02422	0.007171	0.0417
**CIP**	Wave 2	Urbanization	0.014088	0.01004	0.01826
**CIP**	Wave 2	Wealth	0.477119	0.30323	0.65592
**CIP**	Wave 2	Pop. Dens.	-0.150957	-0.21986	-0.08591
**CIP**	Wave 2	Pop. Age	0.081219	0.04883	0.11497
**CIP**	Wave 2	No Travel	-0.308187	-0.5306	-0.08334
**CIP**	Wave 2	Distance	0.035793	-0.15537	0.22363
**CIP**	Wave 2	In-Degree	0.006341	-0.01011	0.02211
**CIR**	Wave 1	Urbanization	0.01251	0.007893	0.01673
**CIR**	Wave 1	Wealth	0.63664	0.442528	0.83091
**CIR**	Wave 1	Pop. Dens.	-0.12855	-0.200722	-0.05848
**CIR**	Wave 1	Pop. Age	0.04736	0.010499	0.08469
**CIR**	Wave 1	No Travel	-0.15135	-0.391388	0.08445
**CIR**	Wave 1	Distance	-0.09669	-0.293352	0.10039
**CIR**	Wave 1	In-Degree	0.02017	0.002489	0.03662
**CIR**	Wave 2	Urbanization	0.01379	9.74E-03	0.01799
**CIR**	Wave 2	Wealth	0.449182	2.69E-01	0.6358
**CIR**	Wave 2	Pop. Dens.	-0.161373	-2.29E-01	-0.09159
**CIR**	Wave 2	Pop. Age	0.07873	4.43E-02	0.11337
**CIR**	Wave 2	No Travel	-0.269283	-4.95E-01	-0.03646
**CIR**	Wave 2	Distance	0.02101	-1.66E-01	0.20443
**CIR**	Wave 2	In-Degree	0.004022	-1.31E-02	0.02024

Supplementary Table 4. Correlations between district-level COVID-19 metrics and Facebook movement data-derived in-degree metrics in India. Higher and significant correlation is seen for cumulative incidence, likely due to a population effect. Population normalized metrics show weak or no correlation.

**Correlation between COVID-19 Metrics and Facebook mobility across districts.**	
**Variable**	**Spearman's Coefficient**
Cumulative Incidence (1st Wave)	0.72*
Cumulative Incidence (2nd Wave)	0.68*
Cumulative Proportion (1st Wave)	0.20*
Cumulative Proportion (2nd Wave)	0.10*
Cumulative Rate (1st Wave)	0.10
Cumulative Rate (2nd Wave)	0.04
* Significant at 0.01 level	

Supplementary Table 5. Regression results for COVID-19 severity ratio with state-level fixed effects.

	Log(Severity Ratio)		
	(1) OLS	*(2) Spatial Lag*	*(3) Spatial Error*
Urbanization	0.0003 (0.002)	0.001 (0.002)	0.001 (0.002)
Wealth	-0.202*** (0.073)	-0.173** (0.069)	-0.187** (0.075)
Log(Population Density)	-0.007 (0.036)	-0.012 (0.033)	-0.033 (0.037)
Population Age	0.047*** (0.014)	0.036*** (0.014)	0.036** (0.016)
Log(No Travel)	-0.105 (0.093)	-0.063 (0.088)	-0.103 (0.094)
Log(Distance)	0.115 (0.079)	0.122 (0.075)	0.109 (0.081)
Log(In-Degree(2nd Wave) / In-Degree(1st Wave))	-0.007 (0.008)	-0.010 (0.007)	-0.010 (0.007)
Constant	-1.723*** (0.559)	-1.490*** (0.531)	-1.269** (0.593)
State Fixed effects?	Yes	Yes	Yes
Rho	-	0.34***	-
Lambda	-	-	0.42***
Observations	636	636	636
R^2	0.407	0.444	0.449
Adjusted R^2	0.367		
Log Likelihood		-280.099	-276.935
$sigma^2$		0.138	0.135
Akaike Inf. Crit.		646.199	639.871
Residual Std. Error	0.401 (df = 595)		
F Statistic	10.195*** (df = 40; 595)		
Wald Test (df = 1)		48.070***	70.625***
LR Test (df = 1)		41.127***	47.455***
Note:	*p**p***p<0.01; Nagelkerke R^2 reported for spatial models; Standard errors are heteroskedasticity-corrected		

Supplementary Table 6. Summary statistics of all the variables considered in the study.

Variable	N	Mean	St. Dev.	Min	Max
**Incidence Proportion (Wave 1)**	639	7,472.436	10,292.350	255.806	111,007.400
**Incidence Proportion (Wave 2)**	636	14,353.180	15,039.100	769.074	111,761.100
**Incidence Rate (Wave 1)**	639	31.788	37.360	1.464	349.158
**Incidence Rate (Wave 2)**	636	116.658	121.039	6.153	963.458
**Severity Ratio**	636	4.520	3.469	0.465	47.350
**R_0 (Wave 1)**	639	1.192	0.137	0.867	2.610
**R_0 (Wave 2)**	636	1.415	0.204	1.037	2.481
**R_t (Wave 1)**	620	1.226	0.109	0.488	1.646
**R_t (Wave 2)**	617	1.478	0.189	1.132	2.728
**Urbanization**	640	29.788	16.948	0.290	93.763
**Average Wealth**	640	-0.001	0.597	-1.137	1.477
**Population Density**	640	1,091.851	3,535.826	1.847	41,618.150
**Average Age**	640	30.076	2.624	22.878	39.788
**% No Travel to Work**	640	34.716	10.349	7.457	68.571
**Average Commute Distance**	640	5.651	1.853	1.231	13.827
**% Travel to Work (On foot)**	640	25.857	8.651	10.218	60.495
**% Travel to Work (Bicycle)**	640	12.399	7.944	0.116	33.117
**% Travel to Work (Motorcycle)**	640	10.493	6.069	0.318	35.670
**% Travel to Work (Car)**	640	2.291	2.353	0.319	19.296
**% Travel to Work (Taxi)**	640	2.424	2.040	0.104	15.714
**% Travel to Work (Bus)**	640	8.985	7.534	0.265	35.037
**% Travel to Work (Train)**	640	1.903	2.629	0.023	28.998
**% Travel to Work (Water Transport)**	640	0.242	0.851	0.000	13.078
**% Travel to Work (Other)**	640	0.690	0.585	0.078	10.434
**In-Degree (Wave 1)**	639	25,328,678.000	42,537,845.000	1.000	506,021,212.000
**In-Degree (Wave2)**	636	13,700,927.000	32,946,809.000	1.000	352,868,895.000
